# Autopsies at Central Park Menagerie

**Published:** 1887-04

**Authors:** 


### CASE DEPARTMENT.

### AUTOPSIES AT CENTRAL PARK MENAGERIE.

TOQUE MONKEY—*Macacus pileatus*, *Hab.* Ceylon. Male. Fairly nourished.

I. EXTERNAL EXAMINATION.—Fur absolutely swarming with nits and dead pediculi. The parasites are very small—about ½ line in size—but have the same general appearance as pedic. vestamenti hominis. A few ecchymoses and scratch marks on abdomen, neck, etc.

II. INTERNAL EXAMINATION.—(*a*) *Spinal Canal*—normal.

(*b*) *Cranium.* Surface of cortex studded with minute hemorrhages. Ruptures of the vessels of the pia in numerous places. The effused blood lies under the pia along the course of the vessels. The left occipital lobe is entirely covered with a layer of blood. This effusion extends round over base of brain—and completely covers the pons ; and surrounding the medulla just below this is a considerable hemorrhage. A number of minute hemorrhages in the corpus callosum. Brain substance otherwise normal. Medulla shows also a large number of small sanguineous effusions.

(*c*) *Thoracic Cavity.—Lungs—Œdematous and Congested.*—Both lungs are filled with a large number of pin-head-sized transparent grayish round nodules, mostly situated under pleura. They are hard, and can be "shelled" out of their capsules easily. *Heart.*—General thickening of parietal and visceral pericardium; numerous ecchymoses upon both layers. Muscle distinctly soft and yellow. In the heart substance are six hard, yellow nodules, pin-head-sized, contents cheesey. At base of heart is one the size of a large pea ; its centre is fluid—and is similar to the purulent collections found elsewhere. *Bronchial Glands*—enlarged, soft, and of a dark, reddish-brown color.

(*d*) *Abdominal Cavity—omentum*—fatless. Glands enlarged, red and soft ; one is bean-sized, with purulent centre. *Peritoneum.*—A few moderately firm old adhesions between the intestinal coils. In the visceral layer of the abdominal peritoneum, left side, three cheesy small-pea-sized masses. *Liver.*—On anterior surface of left lobe, a large bean-sized superficial abscess that broke in handling, letting out a considerable amount of greenish-yellow pus. The entire organ is one mass of abscesses, varying from a pea-size to one inch in diameter. Very little liver tissue is left. The more superficial abscesses have caused considerable perihepatitis, with adhesions to neighboring organs, stomach, intestines, etc. *Spleen.*—Studded with large and small cheesy masses and abscesses 1 line to ¼ inch in diameter. *Pancreas.*—Two purulent cheesy masses, each ⅓ inch in diameter. *Retroperitoneal glands.*—All enlarged, dark, soft. Some are purulent. *Kidneys.*—Pale and yellow ; cortical workings very indistinct. Both organs are studded with large pin-head-sized purulent nodules; some are surrounded by a distinctly inflammatory and hemorrhagic zone of tissue. Pelvis of left kidney is filled with blood. *Bladder.*—Peritoneal surface ecchymosed. Substance contains three yellow purulent pea-sized masses. Mucus membrane shows a number of minute and several large ecchymoses. *Stomach.*—Mucus membrane studded with hemorrhages, some ¼ inch in size. *Intestines, Small.*—Serous surface ecchymosed in parts. In four places, purulent collections in the intestinal walls. *Large.*—The entire large gut is intensely congested. Its substance contains a large number of pea-sized abscesses and smaller blackish-brown nodules which are yet hard. Mucus membrane normal. *Rectum.*—Is bound firmly by adhesions to the large abscess to be next described; its mucus membrane is intensely inflamed—but there is no ulceration. No connection between abscess and gut.

A large fusiform swelling almost entirely fills the true pelvis and the lower third of the abdominal cavity. It lies in the median line along the spinal column, behind the peritoneum; it is pear-shaped, with apex up, and is tense. It contains over two ounces of greenish-yellow pus. Abscess walls are ragged and irregular, and at least a ¼ inch thick. Special and pelvic bones and ligaments normal. On each side it lies over the Psoas-Iliacus muscles—but is not in their sheath. On the left side it covers and is adherent to the rectum—but does not communicate with it. *Joints.*—normal. *Membrane, &c.*—no tubercles anywhere. *Cause of Death.*—Œdema and congestion of lungs. *Diagnosis.*—pyæmia secondary to large retroperitoneal pelvic abscess. Secondary abscesses of liver, spleen, peritoneum, intestinal wall, lungs, kidneys, or diffuse inflammation of pericardium, gastro-intest. mucus.

MACAQUE MONKEY *Macacus Cynomolgus.* *Hab.* India. Female. Dead 12 hours.

I. EXTERNAL EXAMINATION.—Moderate emaciation. Rigidity marked.

II. INTERNAL EXAMINATION.—(*a*) *Thoracic cavity—left lung.*—A few old adhesions. Moderate œdema. *Right lung.*—The entire root of the lung is encircled by a dense walnut-sized nodule, binding it to the posterior chest wall and involving the posterior thoracic glands. Size, 1½ x ¾ inch, with long axis, longitudinal. Nodule is distinctly circumscribed by a dense connective tissue envelope. The whole is a large, cheesy, degenerated inflammatory centre of old date. Thoracic glands enlarged throughout, some cheesy. In the right pleura, diaphragmatic, mediastinal and costal, are fifteen hard, white and prominent nodules, from pin-head to large pea-size; the largest are pigmented. They are all subpleural, cheesy deposits, encapsulated, like the larger nodule. Some are situated upon the ribs at their posterior angle, some along their anterior curves, and others again in the mediastinal and diaphragmatic pleura. Moderate œdema of lung. Marked anæmia of all thoracic organs except heart. No tubercles. *Heart.*—Markedly congested. No tubercles. *Pericardium.*—normal. *Larynx.*—Normal.

(*b*) *Abdominal Cavity—Liver.*—Entire surface studded with pin-point and pin-head sized, grayish-white, transparent nodules. The whole organ is crowded with tubercles—almost all evidently of recent origin. No cheesy masses. *Spleen.*—Enlarged. Moderately studded with tubercles. Five large, pea-sized, white, soft, cheesy masses project prominently from its surfaces. None are encapsulated, and most of them show an area of congestion around them, and enlarged blood-vessels running over their surface. *Pancreas.*—Normal. *Omentum.*—Almost fatless. Some dozen large, pin-head-sized, hard, white nodules, similar to those of left pleural cavity. *Mesentery.*—Glands enlarged and pigmented throughout. A few tubercles. *Peritoneum.*—A few cheesy nodules in the parietal peritoneum. No adhesions. *Abdominal lymphatic glands* are all enlarged. Perfect chains of them accompany the vessels and run along the attached border of the gut, especially along the colon and cæcum. *Intestines.*—Normal. *Kidneys.*—Absolutely white and bloodless, fatty. A few distinct tubercles in their capsules and substance. *Supra renal capsules, uterus and ovaries, bladder and stomach*—anæmic. The entire body, except the heart, was markedly anæmic, and the blood present was thin and watery to a very noticeable degree. No coagula in the large vessels.

*Diagnosis.*—Acute general tuberculosis, secondary to caseous degeneration of most of inflammatory products at the base of the lung.

WEEPING CAPUCHIN *Cebus capucinus.* *Hab.* Brazil. Male.

I. EXTERNAL EXAMINATION.—Moderate emaciation.

II. INTERNAL EXAMINATION.—(*a*) *Cranium.*—normal. *Cord.*—normal.

(*b*) *Thorax—Left cavity.*—A number of recent pleural adhesions. Entire lung solidified ; general lobular pneumonia ; œdema and congestion of non-affected parts. The solidified patches are solid and white ; a few of the oldest and largest have white, cheesy nodules in their centres. *Right Cavity.*—Same conditions. A small amount of clear serum also. *Heart.*—*Pericardial sac* contains two drachms of clear serum. A fibrous clot four inches long, and containing a considerable amount of red globules in its meshes, is attached to the R. appendix auricular, and hangs down into the pericardial cavity. *Parietal pericardium.*—Normal. *Visceral pericardium.*—Rough in places, with spots of adherent lymph. *Heart muscle and valves, &c.*—Normal. *Posterior mediastinal glands.*—Are the size of large beans and filled with softened, cheesy matter. They are continuous above with the curvial glands, which are in the same condition, though not so large. The entire glandular mass is imbedded in firm, fibrous connective tissue, so that the whole forms a large tumor, three inches long by one inch in breadth and in thickness. The œsophagus and trachea pass through the centre of this mass.

(*c*) *Abdomen.*—Very little fat. *Liver.*—Fatty and congested, enlarged *Pancreas.*—Considerable number of pin-point hemorrhages in its tissue. *Spleen.*—Half larger than normal ; dark red and softened. *Abdominal lymphatic glands.*—Enlarged throughout, dark purplish-red in color, and soft. Lymphatic glands throughout entire body in same condition.

*Kidneys.*—softened. *Intestines.*—Fragments of tape worm (2 heads). Both belong to second variety described before.

*Diagnosis.*—Chronic catarrhal pneumonia and peribronchitis, subacute pleurisy, sero-fibrous pericarditis, mediastinitis fibrosa, general lymphadinitis, fatty infiltration and congestion of liver, tænia.

*Cause of death.*—Exhaustion.

WEEPER CAPUCHIN (*Cebus capucinus*). *Hab.* Brazil.

I. EXTERNAL EXAMINATION.—Moderately well nourished.

II. INTERNAL EXAMINATION.

(*a*) *Cranium and Spinal Column.*—*Brain*—general congestion. Vessels at base atheromatous.

(*b*) *Thorax.*—*Lungs*—*Left.*—General congestion and œdema. Scattered nodules of chronic peribronchitis. Spots of ablutosis. Base of lung acutely congested. *Right Lung.*—Entire lung solid. Acute congestion and œdema of entire lung. A number of bean-sized nodules of lobular pneumonia. *Pleura.*—normal. *Heart and pericardium.*—Normal.

(*c*) *Abdomen.*—*Omentum*—is one-third inch thick and contains an immense amount of fat. The whole forms a large mass of fat, in which omentum, intestines and all the abdominal organs are enveloped. *Liver.*—Enlarged, congested and fatty. *Spleen*, *Pancreas*, *Kidneys*—normal. *Intestines*, *Supra renals*, *Bladder*—normal.

*Diagnosis.*—Congestion of the brain, œdema and congestion of lungs, peribronchitis, chronic catarrhal pneumonia, congested and fatty liver.

*Cause of death.*—Apuvea from œdema pulmonum.

WILLIAM S. GOTTHEIL, M. D., New York.

II. RUPTURE OF MESENTERIC ARTERY.—Subject, a bay Shetland pony (foal). The owner, a farmer, noticed that the animal appeared dull, was very weak, and refused to eat. He gave him a dose of ginger, and about half an hour later found him dead.

The post-mortem was made about ten hours after death. The abdomen was greatly distended. On making an incision through the wall, the cavity found to contain a large amount of partially clotted blood, about two large pails full in all. No peritonitis. Intestines appeared normal in situ. The great omentum deeply stained, free and shreddy. There was an aneurism about as large as a hen's egg in the anterior mesenteric artery, just as it is given off from the aorta. At the bifurcation of the right branch there was a clot of blood about the size of an apple, dark in color and somewhat adherent to the mesentery and vessels. There were no necrosed parts of the intestine. The other organs of the body were healthy.

HEMORRHAGE AT THE BASE OF THE BRAIN.—Subject, a black gelding, 1500 pounds weight and 9 years old, the property of a large cartage company in this city.

*History.*—The horse was doing his usual work, and apparently in his usual health. He was in the habit of balking. Just before his death he stopped suddenly and refused to move. The driver plied the whip. The horse reared, fell backwards and struck his head against the curbstone, expiring almost immediately.

*Autopsy.*—Made fifteen hours after death. There were no external lesions to be seen; no bruising of the tissues about the pole; no fractures to be detected. On opening the abdomen the contents were found to be normal, the thoracic organs healthy, both auricles empty, a small amount of blood in ventricles of bright red color and not clotted; valves normal, blood vessels normal. On removal of the brain the meninges were seen to be considerably congested. At the base a large blood clot extended from one and one-half inches in front of the optic chiasma anteriorly, to the anterior border of the pons varolii posteriorly, and was bounded lateraly by the external borders of the crura cerebri. Branches extended along the course of the vessels to the border of the cerebral hemispheres, and upwards along the course of the choroid plexuses to the lateral ventricles.

This clot lay between the pia mater and the substance of the brain.

There was a second clot extending from the medulla some two or three inches down the cord.

The lesions could not be traced. The substance of the brain appeared normal.

Rupture of the Coronary Artery.—The subject was a dark bay gelding, about 15.1 hands high, 12 years of age, and of 1,000 pounds weight, with a trotting record of 2.19 without weights. He dropped dead while being jogged on the track at a six or seven-minute gait.

*Post-mortem* examination made within three hours from time of death. There were no abrasions of the skin; no fractures or dislocations. General condition good. Muscles hard, but perhaps a little too much adipose tissue for a trotter; no abnormal contents in the abdominal cavity; no peritonitis; intestines in situ apparently normal; other abdominal organs normal. On slitting up the intestine, there was seen to be some thickening of the mucous membrane of the ilium, with small hemorrhagic points here and there; the membrane in other parts of the intestine normal.

*Thorax.*—Upon the removal of the sternum, no fluid was present in the pleural sacs. There was an old fracture of the ninth rib on the right side in the lower part of its upper third. This was covered with an organized pleuritic exudate, but there was no adhesion to the pleural surface of the lung. This membrane was about the size of one's hand. The pericardium was greatly distended with a fluctuating, lumpy mass. On opening the sac, a quantity of clotted and partially clotted blood escaped. On enlarging the opening, the sac was found to contain some two or three quarts of clotted blood; the pericardium was normal in appearance; heart muscle firm and of normal color; lungs crepitant, dark in color; aorta empty; veins distended; pulmonary artery filled with buffy clot, not adherent. Each lung was removed separately. Heart, pericardium and twelve inches of the aorta removed together.

The clot extended for some six inches down the aorta beneath the outer coat. All the chambers of the heart were empty; walls firm; muscle substance normal; no endocarditis; no valvular lesions; walls of the aorta firm and healthy, so far as the eye could detect; no aneurism; no atheroma. There was a rupture at the base of the coronary artery, close to the sinus of Valsalva, about half an inch in length. The edges were a little ragged, but the walls seemed healthy. The brain was very well developed and of firm consistence; the cerebellum particularly well developed; the arteries at the base healthy.

A. W. Clement, V. S.,
*Montreal Veterinary College.*

